# Supplementary figures and images for: Length-mass relationships of pond macroinvertebrates do not hold between Southern and Northern Europe
Source: PeerJ. 2024 Dec 16;12:e18576. doi: 10.7717/peerj.18576 (PMC11657199; doi:10.7717/peerj.18576)

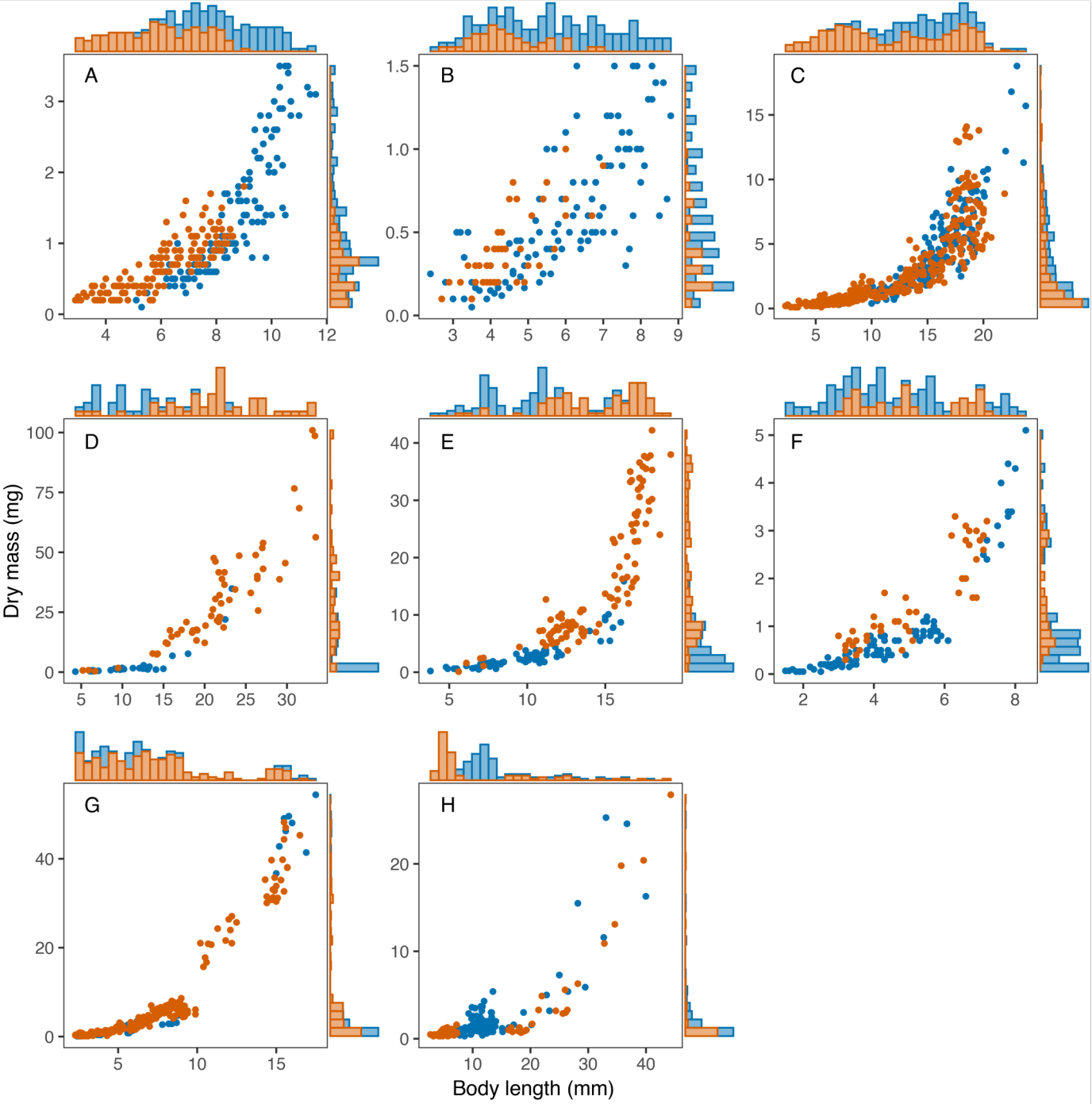

Supplement: Supplemental Information 1 — Scatterplots of the length-mass relationships in Cloeon (A), Caenis (B), Coenagrionidae (C), Aeshnidae (D), Sympetrum (E), Corixidae (F), Notonecta (G), and Dytiscidae (H). Histograms of lengths and masses are plotted parallel to the respective axes. Length-mass relationships from Portugal are in red and from Sweden in blue. [file peerj-12-18576-s001.pdf]

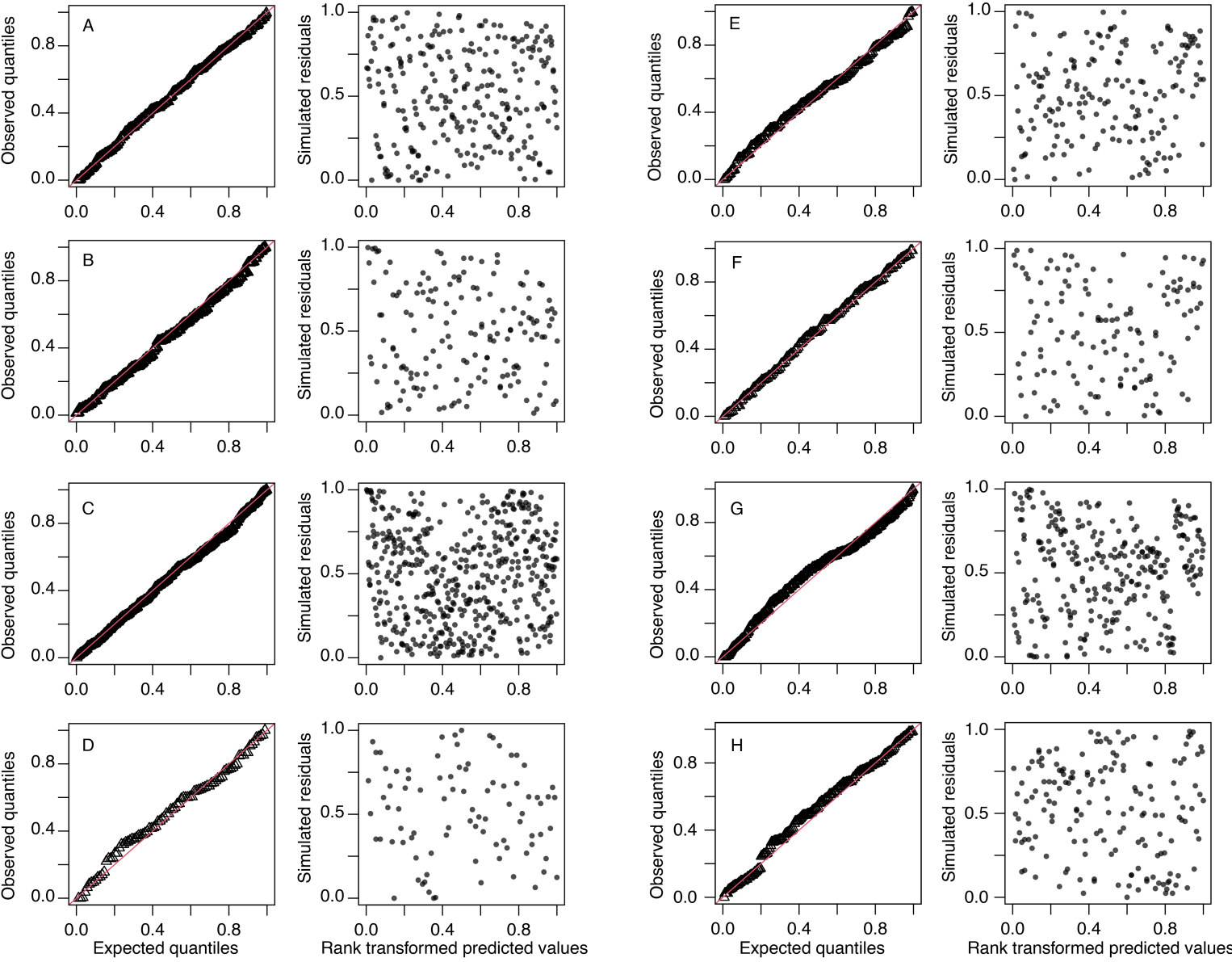

Supplement: Supplemental Information 2 — Diagnostic plots of residuals from the LMMs testing the length-mass relationships in Cloeon (A), Caenis (B), Coenagrionidae (C), Aeshnidae (D), Sympetrum (E), Corixidae (F), Notonecta (G), and Dytiscidae (H). Quantile-quantile plots (left) are shown alongside residuals vs fitted plots (right) for each model. [file peerj-12-18576-s002.pdf]
