# Supplementary material for: Length-mass relationships of pond macroinvertebrates do not hold between Southern and Northern Europe
Source: PeerJ. 2024 Dec 16;12:e18576. doi: 10.7717/peerj.18576 (PMC11657199; doi:10.7717/peerj.18576)
Supplement: Supplemental Information 4 — The raw data shows all individuals of aquatic macroinvertebrates which were sampled, determined and processed in the laboratory for measurements of body length and mass. These individuals were used for estimating length-mass relationships, comparisons and statistical analysis. [file peerj-12-18576-s004.docx]

| **Taxa** | **Species** | **Type of measurement** | **Equation** | **Source** |
| --- | --- | --- | --- | --- |
| **PORTUGAL** |  |  |  |  |
| Amphipoda | *Corophium multisetosum* | head length (LH) | AFDW = 1.7671xLH^3.241^ | Cunha, Moreira & Sorbe (2000) |
| Amphipoda | *Echinogammarus meridionalis* | maximum body length (BL) | DM=0.1633BL – 0.02606 | Gama (2014) |
| Trichoptera | *Schizopelex festiva* | anterior case opening (CO) | DM = 0.5177e^(0.0961* CO)^ |  |
| Amphipoda | *Talitrus saltator* | cephalic length (CL) | AFDW = 0.00261025CL^3.79929^ | Marques et al. (2003) |
|  |  |  | AFDW = 0.023215CL^2.87104^ |  |
|  |  |  | AFDW = 0.024378CL^2.85113^ |  |
| Amphipoda | *Ampithoe valida* | cephalic length (CL) | W = 0.00085xCL^3631^ | Pardal et al. (2000) |
| Amphipoda | *Gammarus insensibilis* | body length |  | Lopes (2014) |
| Amphipoda | *Melita palmat* | body length |  |  |
| Amphipoda | *Microdeutopus gryllotalpa* | body length |  |  |
| Diptera | Chironomidae | body length |  |  |
| class Gastropoda | *Physa acuta* | shell length |  |  |
| class Polychaeta | *Streblospio shrubsolii* | body length |  |  |
| class Bivalvia | 25 species | shell length |  | Gaspar, Santos & Vasconcelos (2001) |
| class Bivalvia | *Chamelea gallina* | shell length | W = -3.193 + 2.797(length) | Rufino et al. (2006) |
| class Bivalvia | *Chamelea striatula* | shell length | W = -3.689 + 3.077(length) |  |
| class Bivalvia | 20 species | shell length |  | Vasconcelos et al. (2018) |
| Decapoda | *Palaemon longirostris* | carapace length (CL) | Log W = -3.005 + 2.884 Log CL | Cartaxana (2003) |
|  |  | carapace length (CL) | Log W = -3.112 + 3.008 Log CL |  |
|  |  | total body length (TL) | Log W = -4.603 +3.039 Log TL |  |
|  |  | total body length (TL) | Log W = -4.494 +2.929 Log TL |  |
| Decapoda | *Procambarus clarkii* | total length (L) | w =1.2 E^-5^ L^3.2^ | Correia (2001) |
| class Gastropoda | *Hydrobia ulvae* | shell height | Biomass = 0.0564 x 2.2381 | Lillebø, Pardal & Marques (1999) |
| Isopoda | *Cyathura carinata* | cephalic length (CL) | AFDW = 0.0190549*CL^2.71815^ | Marques et al. (1994) |
| Mysida | *Mesopodopsis slabberi* | total length (TL) | LnDW = 3.0298 x LnTL -6.0229. | Azeiteiro, Jesus & Marques (1999) |
| Phyllodocida | *Nereis diversicolor* | jaw length (JL) | log_10_AFDW (g) = 2.213 x log_10_JL – 2.088 | Abrantes, Pinto & Moreira (1999) |
|  |  |  | log_10_AFDW (g) = 2.782 x log_10_JL – 2.284 |  |
| Trichoptera | *Lepidostoma hirtum* | distance between eyes (ED) | AFDW = 0.0409*e^3.451*ED^ | Azevedo-Pereira, Graça & González (2006) |
| Trichoptera | *Limnephilus atlanticus* | case length, case opening width, body length, head length, interocular distance |  | Balibrea et al. (2017) |
| Trichoptera | *Sericostoma vittatum* | length of the anterior opening of the case (Lc) | ln Dw = (Lc – 1.664)/0.599 | Canhoto (1994) |
| Trichoptera | *Sericostoma vittatum* | head width (HW) | M = 0.734 HW^3.5211^ | González & Graça (2003) |
|  |  |  |  |  |
| **SWEDEN** |  |  |  |  |
| Amphipoda | 3 species | total length or body width |  | Eklöf et al. (2017) |
| Bivalvia | 3 species | length from anterior to posterior side |  |  |
| Gastropoda | 5 species | height along the central shell axis |  |  |
| Insecta | 3 species | total length |  |  |
| Arthropoda | *Asellus aquaticus* | body length | y = 2.65x-1.84 | Perkins et al. (2010) |
| Plecoptera | *Nemoura cinerea* | body length | y = 1.18x-0.76 |  |
| Trichoptera | *Sericostoma personatum* | straight line linking front to back along the concave surface of its protective case | y = 1.82x-1.03 |  |
|  | 44 species |  |  | Rumohr, Brey & Ankar (1987) |

Sources:

Abrantes A, Pinto F, Moreira MH. 1999. Ecology of the polychaete *Nereis diversicolor* in the Canal de Mira (Ria de Aveiro, Portugal): Population dynamics, production and oogenic cycle. *Acta Oecologica* 20:267-283 DOI: 10.1016/S1146-609X(99)00139-3.

Azeiteiro UM, Jesus L, Marques JC. 1999. Distribution, population dynamics, and production of the suprabenthic mysid *Mesopodopsis slabberi* in the Mondego Estuary, Portugal. *Journal of Crustacean Biology* 19:498-509 DOI: 10.2307/1549259.

Azevedo-Pereira HVS, Graça MAS, González JM. 2006. Life history of *Lepidostoma hirtum* in an Iberian stream and its role in organic matter processing. *Hydrobiologia* 559:183-192 DOI: 10.1007/s10750-005-1267-1.

Balibrea A, Ferreira V, Gonçalves V, Raposeiro PM. 2017. Consumption, growth and survival of the endemic stream shredder *Limnephilus atlanticus* (Trichoptera, Limnephilidae) fed with distinct leaf species. *Limnologica* 64:31-37 DOI: 10.1016/j.limno.2017.04.002.

Canhoto CMMML. 1994. A decomposição e utilização das folhas de Eucalyptus globulus como fonte alimentar por detritívoros aquáticos. Master thesis, University of Coimbra, Portugal.

Cartaxana A. 2003. Growth of the prawn *Palaemon longirostris* (Decapoda, Palaemonidae) in Mira River and estuary, SW Portugal. *Journal of Crustacean Biology* 23:251-257 DOI: 10.1163/20021975-99990334.

Correia AM. 2001. Seasonal and interspecific evaluation of predation by mammals and birds on the introduced red swamp crayfish *Procambarus clarkii* (Crustacea, Cambaridae) in a freshwater marsh (Portugal). *Journal of Zoology* 255:533-541 DOI: 10.1017/S0952836901001625.

Cunha MR, Moreira MH, Sorbe JC. 2000. The amphipod Corophium multisetosum (Corophiidae) in Ria de Aveiro (NW Portugal). II. Abundance, biomass and production. *Marine Biology* 137:651-660 DOI: 10.1007/s002270000385.

Eklöf J, Austin Å, Bergström U, Donadi S, Eriksson BD, Hansen J, Sundblad G. 2017. Size matters: relationships between body size and body mass of common coastal, aquatic invertebrates in the Baltic Sea. *PeerJ* 5:e2906 DOI: 10.7717/peerj.2906.

Gama AM. 2014. Global anthropogenic threats in Portuguese streams: ecological effects on aquatic macroinvertebrates assessed at different levels of biological organization. D. Phil. Thesis, University of Coimbra.

Gaspar MB, Santos MN, Vasconcelos P. 2001. Weight-length relationships of 25 bivalve species (Mollusca: Bivalvia) from the Algarve coast (southern Portugal). *Journal of the Marine Biological Association of the United Kingdom* 81:805-807 DOI: 10.1017/S0025315401004623.

González JM, Graça MAS. 2003. Conversion of leaf litter to secondary production by a shredding caddis‐fly. *Freshwater Biology* 48:1578-1592. DOI: 10.1046/j.1365-2427.2003.01110.x.

Lillebø AI, Pardal MÂ, Marques JC. 1999. Population structure, dynamics and production of Hydrobia ulvae (Pennant) (Mollusca: Prosobranchia) along an eutrophication gradient in the Mondego estuary (Portugal). *Acta Oecologica* 20:289-304 DOI: 10.1016/S1146-609X(99)00137-X.

Lopes MFL. 2014. Ecological quality assessment in transitional systems. D. Phil. Thesis, Univeesity of Aveiro.

Marques JC, Gonçalves SC, Pardal MA, Chelazzi L, Colombini I, Fallaci M, Bouslama MF, El Gtari M, Charfi-Cheikhrouha F, Scapini F. 2003. Comparison of *Talitrus saltator* (Amphipoda, Talitridae) biology, dynamics, and secondary production in Atlantic (Portugal) and Mediterranean (Italy and Tunisia) populations. Estuarine. *Coastal and Shelf Science* 58:127-148 DOI: 10.1016/S0272-7714(03)00042-8.

Marques JC, Martins I, Teles-Ferreira C, Cruz S. 1994. Population dynamics, life history, and production of *Cyathura carinata* (Krøyer)(Isopoda: Anthuridae) in the Mondego estuary, Portugal. *Journal of Crustacean Biology* 14:258-272 DOI: 10.1163/193724094X00254.

Pardal MA, Marques JC, Metelo I, Lillebø AI, Flindt MR. 2000. Impact of eutrophication on the life cycle, population dynamics and production of *Ampithoe valida* (Amphipoda) along an estuarine spatial gradient (Mondego estuary, Portugal). *Marine Ecology Progress Series* 196:207-219 DOI: 10.3354/meps196207.

Perkins DM, McKie BG, Malmqvist B, Gilmour SG, Reiss J, Woodward G. 2010. Environmental warming and biodiversity-ecosystem functioning in freshwater microcosms: partitioning the effects of species identity, richness and metabolism. *Advances in ecological research* 43:177-209 DOI: 10.1016/B978-0-12-385005-8.00005-8.

Rufino MM, Gaspar MB, Pereira AM, Vasconcelos P. 2006. Use of shape to distinguish *Chamelea gallina* and *Chamelea striatula* (Bivalvia: Veneridae): linear and geometric morphometric methods. *Journal of Morphology* 267:1433-1440 DOI: 10.1002/JMOR.10489.

Rumohr H, Brey T, Ankar S. 1987. *A compilation of biometric conversion factors for benthos invertebrates of the Baltic Sea*. Institut für Meereskunde.

Vasconcelos P, Moura P, Pereira F, Pereira AM, Gaspar MB. 2018. Morphometric relationships and relative growth of 20 uncommon bivalve species from the Algarve coast (southern Portugal). *Journal of the Marine Biological Association of the United Kingdom* 98:463-474 DOI: 10.1017/S002531541600165X.
